# Negative Thermal Expansion over a Wide Temperature Range in Fe-Doped MnNiGe Composites

**DOI:** 10.3389/fchem.2018.00015

**Published:** 2018-02-06

**Authors:** Wenjun Zhao, Ying Sun, Yufei Liu, Kewen Shi, Huiqing Lu, Ping Song, Lei Wang, Huimin Han, Xiuliang Yuan, Cong Wang

**Affiliations:** ^1^Department of Physics, Center for Condensed Matter and Materials Physics, Beihang University, Beijing, China; ^2^Capital Normal University High School, Beijing, China

**Keywords:** Fe-Doped MnNiGe, Heusler alloys, negative thermal expansion, magnetic transition, composites

## Abstract

Fe-doped MnNiGe alloys were successfully synthesized by solid-state reaction. Giant negative thermal expansion (NTE) behaviors with the coefficients of thermal expansion (CTE) of −285.23 × 10^−6^ K^−1^ (192–305 K) and −1167.09 × 10^−6^ K^−1^ (246–305 K) have been obtained in Mn_0.90_Fe_0.10_NiGe and MnNi_0.90_Fe_0.10_Ge, respectively. Furthermore, these materials were combined with Cu in order to control the NTE properties. The results indicate that the absolute value of CTE gradually decreases with increasing Cu contents. In Mn_0.92_Fe_0.08_NiGe/*x*%Cu, the CTE gradually changes from −64.92 × 10^−6^ K^−1^ (125–274 K) to −4.73 × 10^−6^ K^−1^ (173–229 K) with increasing value of *x* from 15 to 70. The magnetic measurements reveal that the NTE behaviors in this work are strongly correlated with the process of the magnetic phase transition and the introduction of Fe atoms could also change the spiral anti-ferromagnetic (s-AFM) state into ferromagnetic (FM) state at low temperature. Our study launches a new candidate for controlling thermal expansion properties of metal matrix materials which could have potential application in variable temperature environment.

## Introduction

Solid materials usually expand during heating while contract during cooling, i.e., positive thermal expansion (PTE). When functional materials are used in variable temperature environment, PTE will reduce the structural stability and safety reliability of precision parts, or even destroy the functional properties of materials (Chen et al., 2015). Thus, zero thermal expansion (ZTE) or controllable thermal expansion materials are highly required and also wildly applied (Wang et al., 2012) in areas such as aerospace, optical instruments, high-precision machining and processing, microelectronic devices, electro-optical sensor, electronic packaging, and so on. It is important to explore new NTE materials, which could be used to combine with PTE materials as thermal-expansion compensators to obtain near-ZTE materials by adjusting their mass ratio. Until now, there are some kinds of material systems with NTE property have been reported, such as ZrW_2_O_8_ (Sun X. J. et al., 2013; Ge et al., 2016), PbTiO_3_-based compounds (Chen et al., 2011), (Bi,La)NiO_3_ (Azuma et al., 2011), antiperovskite manganese nitrides (Sun et al., 2012b; Yan et al., 2012), ScF_3_-based compounds (Greve et al., 2010), La(Fe,Co,Si)_13_ (Huang et al., 2013), MnCoGe-based materials (Zhao et al., 2015), Ca_2_RuO_4_ (Takenaka et al., 2017), and so on. The previous studies have revealed different mechanisms which lead to the NTE behaviors, including phonon model (Li et al., 2002), rigid unit modes (Dove et al., 1995), phase transition mechanism (Xing et al., 2003), electronic valence transfer mechanism (Arvanitidis et al., 2003), magnetic transition mechanism (Sun et al., 2012a), and so on.

Recently, MnM'X (M': Co, Ni, Fe, et al, X: Si, Ge, Sn, et al) Heusler alloys have attracted a lot of attention due to the abundant structural and magnetic properties. In 2013, researchers found that the volume of polycrystalline MnCoSi alloy contracted about 0.2% at 300 K (Barcza et al., 2013). Especially, Zhao et al. (2015) found that MnCo_0.98_Cr_0.02_Ge, bonded with 3–4 wt.% epoxy, undergoes a giant NTE with a linear thermal coefficient of −119 × 10^−6^ K^−1^ from 250 to 305 K during the martensitic structural transition. Furthermore, the result from Lin et al. (2016) indicates that fine-powder Mn_0.98_CoGe can broaden the NTE temperature window to ~135 K (258–393 K) with CTE −79.6 × 10^−6^ K^−1^.

As a typical MnM'X Heusler alloy, MnNiGe undergoes a martensitic structural transition from the hexagonal Ni_2_In-type structure (space group P63/mmc, 194) to orthorhombic TiNiSi-type structure (space group Pnma, 62) at ~470 K during cooling process (Ma et al., 2015), which means that there is a potentially good negative thermal expansion effect in this system. Otherwise, the previous study indicates that the introduction of Fe in MnNiGe alloy can change the magnetic competing relationship in parent phases (Liu et al., 2012). And researchers do have observed NTE property in MnNiGe-based compounds after combining with epoxy resin, i.e., a giant CTE of −60.7 × 10^−6^ K^−1^ over 231–338 K (ΔT = 107 K) and a near-ZTE of 0.6 × 10^−6^ K^−1^ over 175–231 K (ΔT = 56 K) for the sample of Mn_0.94_Fe_0.06_NiGe after mixing with 20 wt.% of epoxy resin (Xu et al., 2017). Unfortunately, the NTE properties of pure MnNiGe-based compounds have not been reported due to the brittle of MnNiGe-based compounds, to our knowledge. Thus, we prepared Fe-doped MnNiGe compounds successfully by solid-state reaction, and observed the giant NTE properties near room temperature (RT) in pure parent alloys. Moreover, the NTE properties were further controlled by combining with commercial Cu. The obtained results in this work broaden the application of Fe-doped MnNiGe alloys.

## Experimental details

Polycrystalline samples of Mn_1−x_Fe_*x*_NiGe (*x* = 0.08, 0.10, 0.16) and MnNi_0.90_Fe_0.10_Ge, Mn_1−x_Fe_*x*_NiGe/Cu (*x* = 0.08, 0.10, 0.16) and MnNi_0.90_Fe_0.10_Ge/Cu composites (hereafter referred as MF*x* (*x* = 8, 10, 16) and NF10, MF*x*/Cu (*x* = 8, 10, 16) and NF10/Cu, respectively) were prepared by solid-state reaction, using Mn, Fe, Ni, Ge, and Cu (4N) powders as raw materials (Deng et al., 2015). Firstly, the stoichiometric amounts of the starting materials (Mn, Ni, Fe, Ge powders) were mixed, then ground about 2 h in a mortar and pressed into pellets by tablet machine. The pellets were sealed in a quartz tube under vacuum (10^−5^ Pa). The quartz tube was sintered in a box furnace at 900°C for 80 h and cooled down to RT. Then, the composites of Mn_1−x_Fe_*x*_NiGe/Cu (*x* = 0.08, 0.10, 0.16), MnNi_0.90_Fe_0.10_Ge/Cu were prepared by mixing Mn_1−x_Fe_*x*_NiGe (*x* = 0.08, 0.10, 0.16), MnNi_0.90_Fe_0.10_Ge and commercial Cu powder with different mass ratio, respectively. After grounding the mixture in an agate mortar for 1 h, the mixture was pressed into rectangular blocks and sealed into quartz tubes in vacuum. Finally, the quartz tubes were sintered in a box furnace at 500°C for 10 h, and then cooled down to RT naturally.

X-ray diffraction (XRD) patterns were obtained from an X' Pert PRO powder diffractometer using Cu K_α_ radiation at (RT), and the XRD data were handled with a software MDI jade 6.0. The linear thermal expansion coefficients of samples were measured from 125 to 475 K by using a Netzsch DIL 402C dilatometer. The instantaneous linear coefficient of thermal expansion α_l_ over certain temperature range for every sample was calculated based on the as-measured thermal strain. The α_l_ is defined as:

(1) $$\begin{array}{r} {\text{α}}_{\text{l}}={\left(1/{\text{L}}_{0}\right)}^{*}\left(\text{dL}/\text{dT}\right) \end{array}$$

where dL and dT are the variations in length and temperature respectively, L_0_ is the initial length at 125 K, dL/L_0_ is the thermal expansion strain (Chen et al., 2015).

The temperature dependence of magnetization curves were measured between 10 and 450 K under zero field-cooling condition (ZFC) in an applied magnetic field of 1,000 Oe using a Physical Property Measurement System (PPMS). A scanning electron microscopy (SEM, NanoSEM430, FEI) was used to characterize the microstructure of composites.

## Results and discussion

### Phase purity and crystal structure

**Figure 1:**
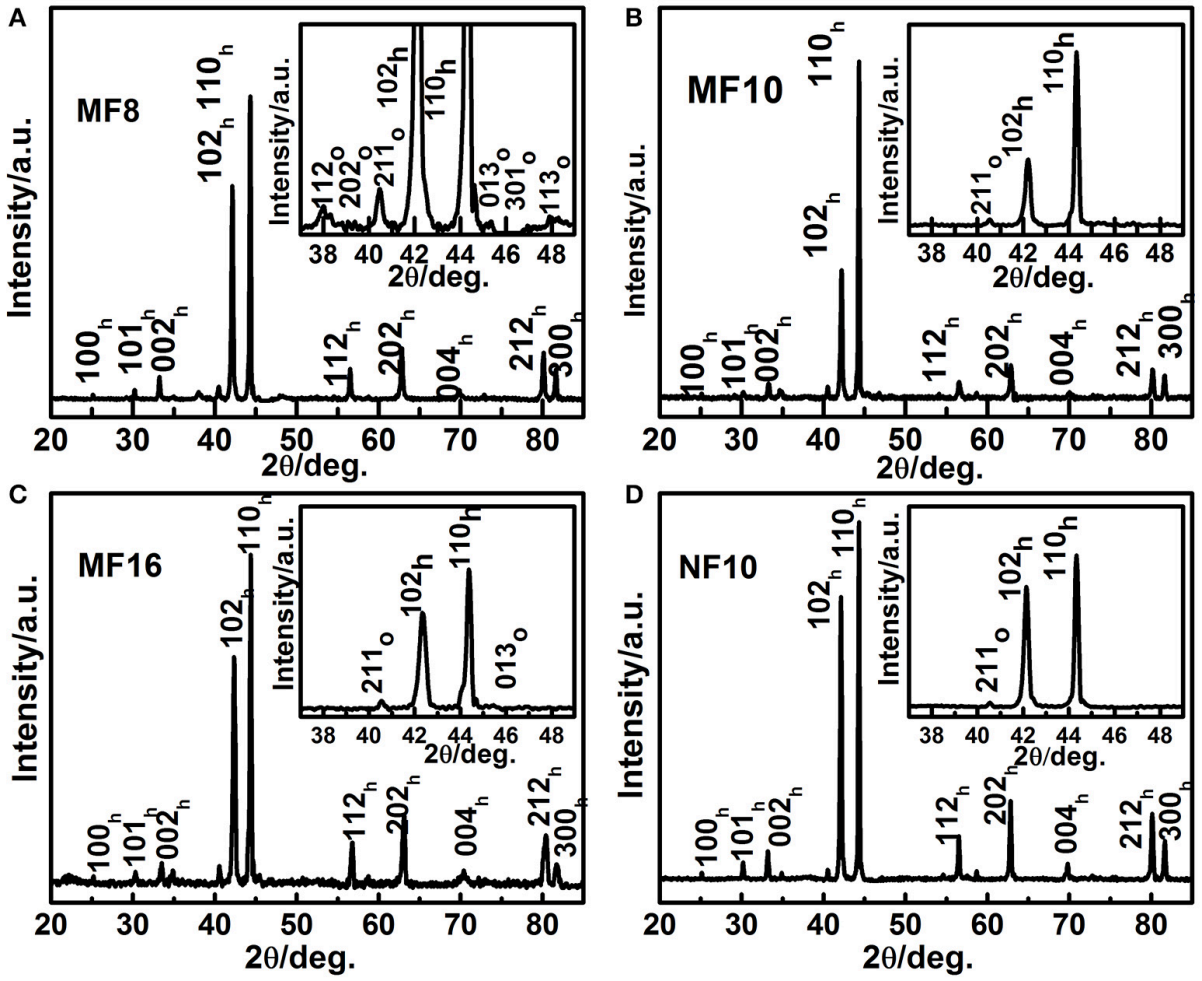
Room temperature X-ray diffraction patterns of **(A)** MF8, **(B)** MF10, **(C)** MF16, and **(D)** NF10.

First, the room temperature XRD patterns of MF8, MF10, MF16, and NF10 are collected to inspect the effect of Fe doping on the crystal structure of MnNiGe alloy. As shown in Figure 1, the mainly diffraction patterns of samples can be indexed using hexagonal Ni_2_In-type structure. Based on XRD data, the lattice parameters (a_h_ and c_h_) of MF8, MF10, MF16, and NF10 are calculated by Rietveld refinement as shown in Figure 1. The results indicate that both a_h_ and c_h_ decrease with increasing Fe doping content at Mn sites because the atom radius of the Mn is larger than Fe. From the enlarged view of XRD patterns in the inset of Figure 1, it is clear that residual orthorhombic TiNiSi-type structure appeared in the main matrix of hexagonal Ni_2_In-type structure for all four samples, the residual mass fractions of orthorhombic phase are calculated to be 14, 4, 4, and 3 wt.%, respectively.

### Thermal expansion properties

**Figure 2:**
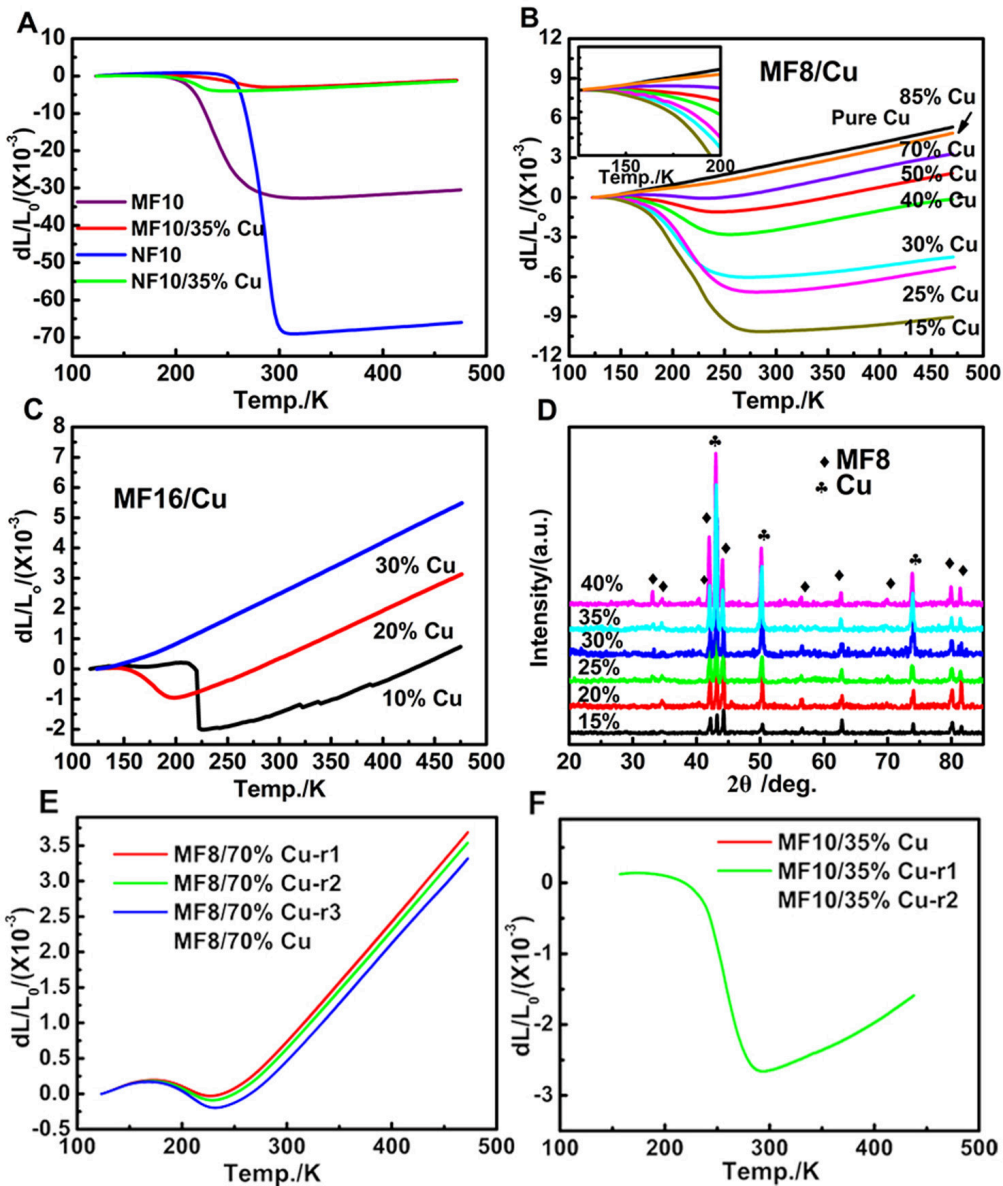
The temperature dependence of dL/L_0_ curves of **(A)** MF10, NF10, MF10/35%Cu and NF10/35%Cu, **(B)** MF8/*x*%Cu (*x* = 15, 25, 30, 40, 50, 70, 85, 100) and **(C)** MF16/*x*%Cu (*x* = 10, 20, 30), where the L_0_ is the length of the sample at 123 K; **(D)** X-Ray patterns of MF8/*x*%Cu composites at RT; the repeated measurement temperature dependence of dL/L_0_ curves of **(E)** MF8/70%Cu and **(F)** MF10/35%Cu.

Linear thermal expansion curves of samples have been recorded from 125 to 475 K in Figure 2. Table 1 lists the thermal expansion values and the related temperature ranges. As shown in Figure 2A, obvious NTE behaviors are observed in MF10 and NF10 near RT, respectively. The average CTE of MF10 reaches to a giant value of −285.23 × 10^−6^ K^−1^ with a wide operation-temperature range of 192–305 K (ΔT = 113 K), while the CTE of NF10 is as large as −1167.09 × 10^−6^ K^−1^ with a temperature range of 246–305 K (ΔT = 59 K). The results indicate that Fe-doped at Ni sites in MnNiGe has a broader temperature range while a larger CTE value can be obtained by Fe doping at Mn sites. Unfortunately, the dL/L_0_ curves of pure MF8 and MF16 have not been obtained due to the brittle of the two samples. By comparison, the CTE of Fe-doping MnNiGe is obviously larger than the reported systems such as ZrW_2_O_8_ (−9.1 × 10^−6^ K^−1^) (Mary et al., 1996), MnCoGe-based compound (−141 × 10^−6^ K^−1^) (Zhao et al., 2015; Lin et al., 2016), and Mn-Co-Ge-In compound (−51.5 × 10^−6^ K^−1^) (Shen et al., 2017).

**Table 1:** CTEs and ΔT of the composites with different Cu mass ratio.

**No**.	**Samples**	**Mass ratio of MF*x*/NF10 (%)**	**CTE(× 10^−6^ K^−1^)**	**Temperature range (ΔT) /K**
1	MF10	100	−285.23	192–305 (113)
2	MF10/35%Cu	65	−26.15	167–290 (123)
3	NF10	100	−1167.09	246–305 (59)
4	NF10/35%Cu	65	−56.73	176–247 (71)
5	MF8/15%Cu	85	−64.92	125–274 (149)
6	MF8/25%Cu	75	−50.23	125–264 (140)
7	MF8/30%Cu	70	−41.99	126–270 (144)
8	MF8/40%Cu	60	−22.53	130–255 (125)
9	MF8/50%Cu	50	−11.32	141–241 (100)
10	MF8/70%Cu	30	−4.73	173–229 (56)
11	MF16/10%Cu	90	1.16	125–215 (90)
			−174.58	216–228 (112)
12	MF16/20%Cu	80	−13.26	125–195 (71)

One of the typical applications for NTE materials is to control thermal expansion behaviors in the form of PTE/NTE composites. In this work, commercial Cu has been selected as matrix metal because of its excellent electronic and mechanic properties and wide usage in the electronic industrial area. Otherwise, in our materials, Fe-doped MnNiGe alloys always collapsed during naturally cooling or the measurement process of CTE, the combination with Cu can also increase the mechanical property of Fe-doped MnNiGe alloys. Therefore, variable mass ratio of MF8/*x*%Cu (*x* = 15, 25, 30, 40, 50, 70, 85, 100), MF16/*x*%Cu (*x* = 10, 20, 30), MF10/35%Cu and NF10/35%Cu compounds were prepared. Figure 2D displays the XRD patterns of the MF8/*x*%Cu composites. All of the diffraction peaks can be indexed as characteristic peaks of either MF8 or Cu and no additional peaks were observed, indicating that no chemical reaction generated between MF8 and Cu during heating treatment in furnace.

The measurements for linear thermal expansion behaviors have been carried out as shown in Figure 2. The amplitudes of the NTE behaviors (ΔL/L_0_) in MF10 and NF10 obviously decrease from 3.18 to 0.40% and 6.95 to 0.32% by introducing 35%Cu, and the CTEs (listed in Table 1) gradually decrease from −285.23 × 10^−6^ K^−1^ (192–305 K) and −1167.09 × 10^−6^ K^−1^ (246–305 K) to −26.15 × 10^−6^ K^−1^ (167–290 K) and −56.73 × 10^−6^ K^−1^ (176–247 K), respectively, as shown in Figure 2A. The linear thermal expansion behaviors of MF8/*x*%Cu and MF16/*x*%Cu are presented in Figures 2B,C).With the increasing of Cu mass ratio *x* for both of the two serials composites, the NTE temperature ranges become narrow, i.e., the decreasing of the ΔT, and the absolute CTE values increase. Obviously, in MF8/*x*%Cu serials composites, a low thermal expansion behavior with a CTE of −4.73 × 10^−6^ K^−1^ in a wide temperature range 173–229 K (ΔT = 56 K) is observed in MF8/70%Cu composite. When the mass ratio of Cu is 30% (sample No. 7 in Table 1), the CTE is −41.99 × 10^−6^ K^−1^ between 126 K to 270 K. The NTE behavior is observed in the temperature range 119–274 K (ΔT = 155 K) for sample MF8/15%Cu, and the corresponding CTE is −64.92 × 10^−6^ K^−1^. In MF16/10%Cu composite, there is a low CTE of 1.16 × 10^−6^ K^−1^ in a wide temperature range 125–215 K (ΔT = 90 K). With further increasing temperature, a sharp decreasing in NTE curve is observed and the corresponding CTE is −174.58 × 10^−6^ K^−1^ (216–228 K). However, the negative thermal expansion disappeared when the mass ratio of Cu increases to 30% in MF16/*x*%Cu composites, this may be due to the NTE temperature region decreases to below 125 K which is the minimum measuring temperature limit. In order to confirm the stability of the NTE behavior in our samples, we measured the thermal expansion properties of MF10/35%Cu and MF8/70%Cu again and the results are very close to the first measurement result, as shown in Figures 2E,F. Therefore, our studies suggest this could be a new alternative for metal matrix composites to achieve near-ZTE materials.

### Magnetic properties

**Figure 3:**
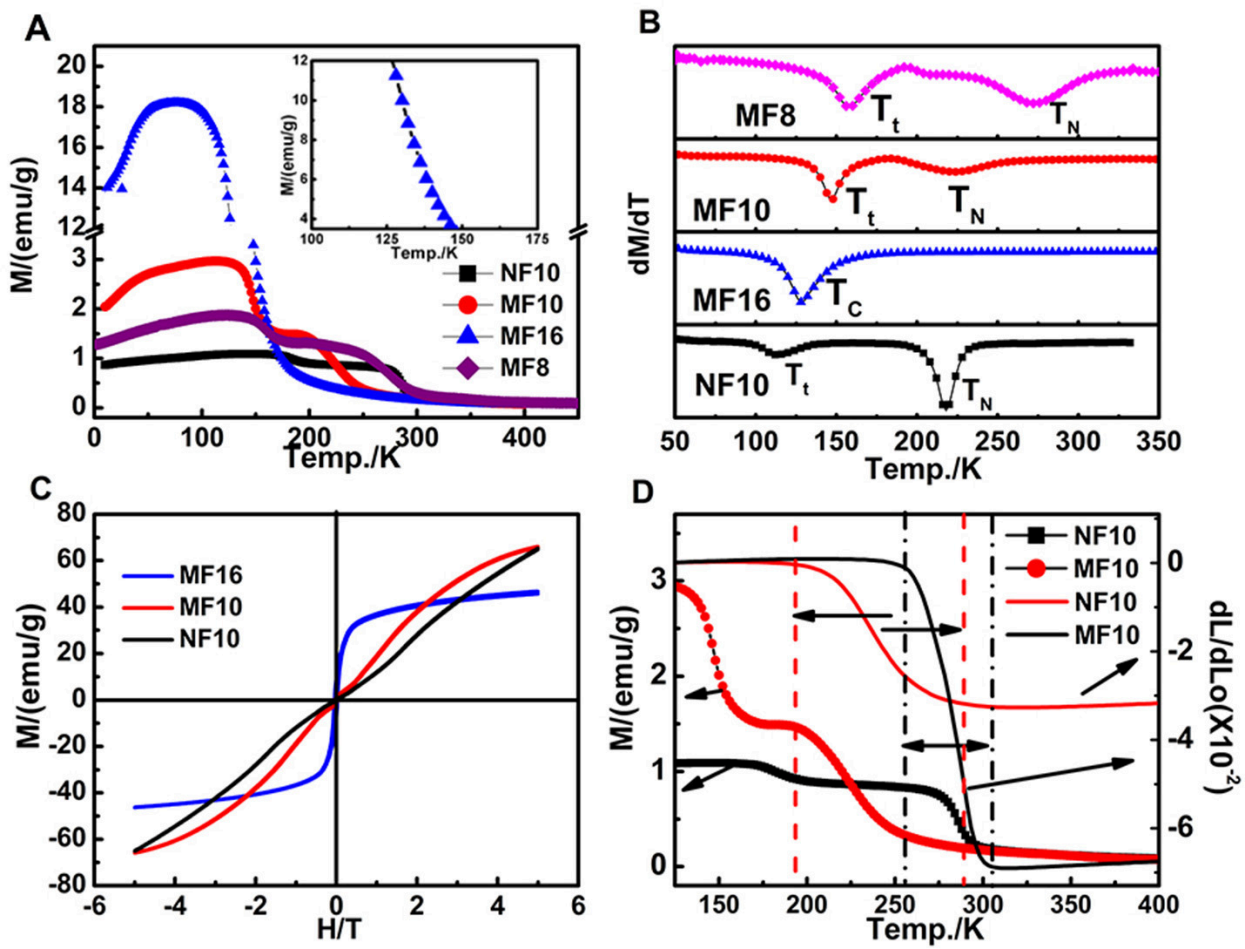
**(A)** M-T curves (ZFC) of MF8, MF10, MF16, and NF10 with external magnetic field of 1000 Oe; **(B)** the related dM/dT-T curves of four samples; **(C)** the isothermal M(H) curves of MF10, MF16, and NF10 at 10 K; **(D)** M(T) curves and dL/L_0_ curves of pure MF10 and NF10 over 125–400 K.

To well understand the thermal expansion property of samples, magnetic properties are further investigated. Temperature dependence of magnetization (M-T) curves of the Fe-doped MnNiGe alloys are first carried out as shown in Figure 3A (the inset presents the breaking part of MF16), and the related dM/dT-T curves are shown in Figure 3B. The M-T curves of MF8, MF10, and NF10 increase slowly first and then drop sharply with temperature increasing, indicating that a magnetic transition occurred. With further increasing temperature, the M-T curves undergo another sharp drop and hence enter into paramagnetic phases (PM). There are two transition points of magnetic properties, which indicates two different ordered magnetic phases exist in MF8, MF10, and NF10. The magnetic transition temperature points (the low transition temperature T_t_ and Néel temperature T_N_) are defined in Figure 3B (Azuma et al., 2011) to be T_t_ = 158 K, 146 K and 112 K, T_N_ = 272, 224, and 218 K for MF8, MF10, and NF10, respectively.

In order to clarify the types of magnetic phases, the isothermal magnetization M(H) curves at 10 K of MF10 and NF10 are carried out in Figure 3C. The M-H curves of MF10 and NF10 tend to be linear behavior with no hysteresis behaviors and near zero macroscopic saturation magnetization in the low magnetic field, indicating typical AFM characters (Song et al., 2008; Sun Y. et al., 2013). The slightly bending of the M-H curves may be caused by a potential magnetic phase transition under external magnetic field. Depending on the previous studies, this AFM phase in low temperature is the typical spiral AFM (s-AFM) (Johnson and Frederick, 1973). In MF10 and NF10, with temperature increasing to T_t_, s-AFM phase transforms to another AFM phase, of which the magnetization is lower than that of s-AFM, as shown in Figure 3A. With further increasing temperature, all of the three samples transit to PM phase at T_N_, successively. However, only one magnetic phase transition is observed in MF16, and the M-H curve shows typical FM characteristics which indicates the magnetic transition of MF16 is a FM-PM transition at Currie temperature T_C_ = 128 K as shown in Figure 3B. In addition, in MF*x* (*x* = 8, 10, 16), with Fe content increasing, the magnetization increased (Figure 3A) and T_t_ or T_N_/T_C_ decreased regularly (Figure 3B), signifying that the introduction of Fe has changed the s-AFM structure into an FM state. Moreover, Figure 3D shows the relationship of dL/dL_0_ and M(T) curves in MF10 and NF10. The black real line and the black dot real line are dL/dL_0_ curve and M(T) curve for MF10, while the temperature range related NTE property has been defined by the perpendicular black dot dash line with a double arrow. Similarly, the red ones are corresponding to NF10. It is clear that the large temperature windows of observed NTE behaviors are in consistent with the magnetization decreasing slowly during the AFM-PM transformation in MF10 and NF10, which indicates a strong relationship between magnetism and thermal expansion properties in these serials compounds, as well the composites.

### Microstructural properties

**Figure 4:**
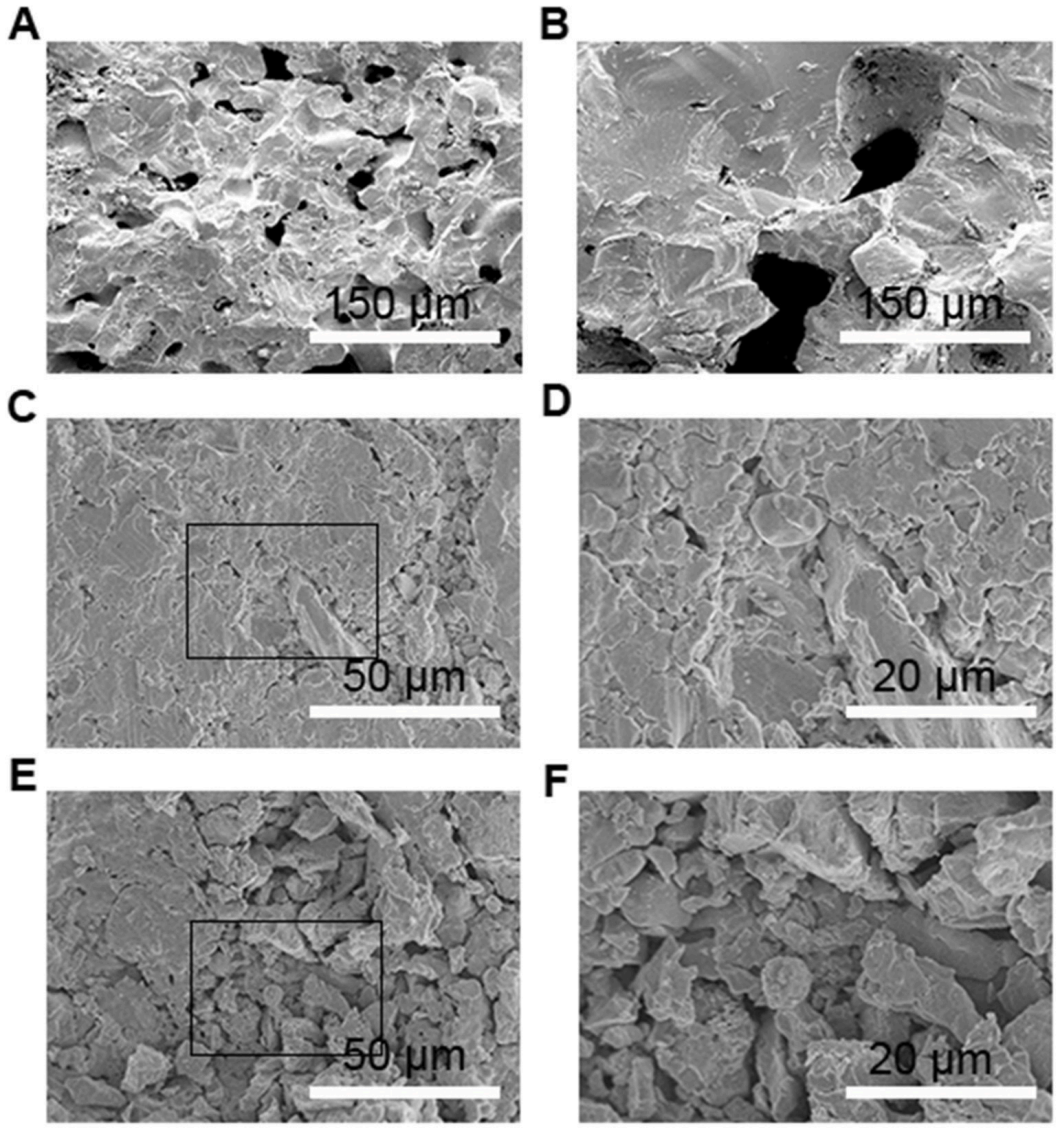
SEM images of **(A)** MF10 and **(B)** NF10, **(C,D)** MF8/70%Cu, **(E,F)** MF10/35%Cu.

**Figure 5:**
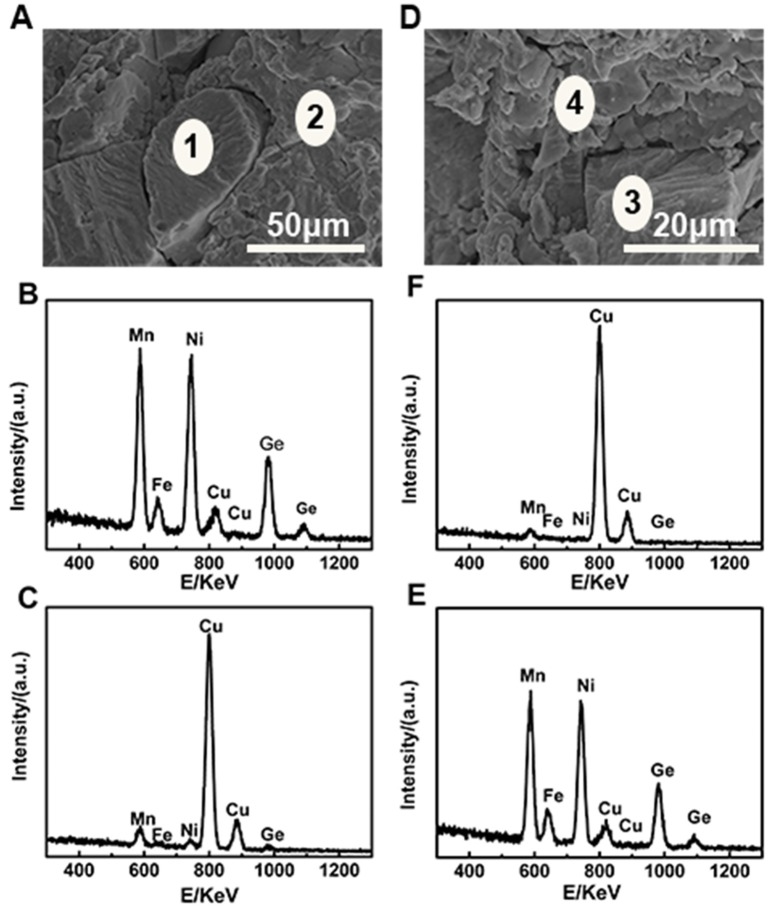
SEM images of **(A)** MF8/70%Cu and **(D)** MF10/35%Cu, respectively and related EDS spectra of **(B,C)** in **(A)** and **(E,F)** in **(D)**, respectively.

The microstructures of pure MF10 and NF10, composites MF8/70%Cu and MF10/35%Cu are carried out and shown in Figure 4. Firstly, in both of MF10 and NF10 samples have massive cavities, and the size of cavities in MF10 is smaller (~28 μm) than that in NF10 in which the size of cavities is as large as 60 μm. Compared with the results of NTE in Figure 2A, large size of cavities sample (NF10) has a larger NTE behavior which indicated that the porosity in pure Fe-doped MnNiGe has positive contribution to the large NTE behaviors. Otherwise, it is clear that two kinds of aggregations and a few holes and cracks can be seen in composites samples. One aggregation consists of small flaky particles with a size ~5 μm, and the other one consists of irregular and granular particles with a larger size (more than 20 μm). Meanwhile, the chemical compositions of these areas are also analyzed by the energy dispersive X-Ray spectroscopy (EDS). We labeled the locations containing different species in Figures 5A,D as areas 1 and 2, areas 3 and 4, respectively. The related EDS of areas 1–2 and areas 3–4 are shown in Figures 5B,C and Figures 5E,F, respectively. As shown in Figure 5, Mn, Ni, Fe, Ge, and Cu peaks are observed in all of the spectra. In MF8/70%Cu, the composition analysis demonstrates that the atomic ratio of area 1 is Mn: Fe: Ni: Ge: Cu = 24.36: 3.77: 35.70: 32.95: 3.22. Combined with Figure 2D, the main component of area 1 is MF8, the small amount of Cu is mainly originating from the adherence during mixed grinding and isothermal heat treatment. The atomic ratio of the area 2 is Mn: Fe: Ni: Ge: Cu = 2.01: 0.22: 0.83: 1.30: 95.62, which indicates the major component of the particles in area 2 is Cu metal. Using the same analysis method, the main component of area 3 is MF10, while the component of area 4 is mainly Cu, as shown in Figures 5E,F. It can be found that the cracks and holes are filled with Cu particles and the composites have a more compact structure with increasing Cu content, i.e., the MF8 is well dispersed in Cu matrix in MF8/70%Cu, where the stability of the composites is guaranteed (Yan et al., 2014). The composites display low thermal expansion or near-ZTE properties due to the competition between the positive expansion property of Cu matrix and the negative thermal expansion properties of Fe-doped MnNiGe alloys when placed in a variable temperature environment.

## Conclusion

In summary, polycrystalline samples of Mn_1−x_Fe_*x*_NiGe (*x* = 0.08, 0.10, 0.16) and MnNi_0.90_Fe_0.10_Ge were prepared by solid-state reaction. Giant negative thermal expansion properties were observed over a wide temperature region in Fe-doped MnNiGe compounds. Especially, the average coefficient of thermal expansion (CTE) for NF10 reaches as large as −1167.09 × 10^−6^ K^−1^ between 246 and 305 K (ΔT = 59 K). The scanning electron microscopy results show that the porosity has positive contribution to the NTE behavior of MF10 and NF10. AFM-PM phase transition, which is correlated with negative thermal expansion (NTE) of the samples, has been characterized by the magnetic measurements. The Mn_1−x_Fe_*x*_NiGe/Cu (*x* = 8, 16) composites with controllable thermal expansion have been successfully prepared by adjusting the mass ratio of Cu. In MF8/*x*%Cu composites, the absolute value of average CTE gradually decreases with increasing matrix Cu content and the CTE reaches to −4.73 × 10^−6^ K^−1^ (173–229 K) with 70%Cu. In addition, the combining with metal Cu also modifies the mechanical performance of Fe-doped MnNiGe compounds. This can be a new candidate for controlling thermal expansion properties of metal matrix materials which may have potential applications in variable temperature environment.

## Author contributions

YS, CW, WZ conceived the idea and designed experiments; WZ, YL, XY, and HH carried out experiments; and KS, HL, PS, and LW analyzed experimental results. All authors wrote and reviewed the manuscript.

### Conflict of interest statement

The authors declare that the research was conducted in the absence of any commercial or financial relationships that could be construed as a potential conflict of interest.
